# Gut microbiota is involved in the exacerbation of adrenal glucocorticoid steroidogenesis in diabetic animals by activation of the TLR4 pathway

**DOI:** 10.3389/fendo.2025.1555203

**Published:** 2025-05-27

**Authors:** Nathalia Santos Magalhães, Amanda Silva Chaves, Beatriz Thomasi, Daniella Bianchi Reis Insuela, Heidi Pauer, Amanda Mendes Rêgo, Cristiane Cassiolato Pires Hardoim, Luis Caetano Martha Antunes, Patrícia Machado Rodrigues e Silva, Marco Aurélio Martins, Vinicius Frias Carvalho

**Affiliations:** ^1^ Laboratory of Inflammation, Center for Research, Innovation and Surveillance in Covid-19 and Health Emergencies, Oswaldo Cruz Institute, Oswaldo Cruz Foundation, Rio de Janeiro, Brazil; ^2^ Department of Molecular Biosciences, University of Kansas, Lawrence, KS, United States; ^3^ National Institute of Science and Technology on Neuroimmunomodulation (INCT-NIM), Oswaldo Cruz Institute, Oswaldo Cruz Foundation, Rio de Janeiro, Brazil; ^4^ Rio de Janeiro Research Network on Neuroinflammation (RENEURIN), Oswaldo Cruz Institute, Oswaldo Cruz Foundation, Rio de Janeiro, Brazil; ^5^ Department of Physiology, Michigan State University, East Lansing, MI, United States; ^6^ Laboratory of Bacteriology Applied to One Health and Antimicrobial Resistance, Oswaldo Cruz Institute, Oswaldo Cruz Foundation, Rio de Janeiro, Brazil; ^7^ National Institute of Science and Technology for Innovation in Diseases of Neglected Populations, Center for Technological Development in Health, Oswaldo Cruz Foundation, Rio de Janeiro, Brazil; ^8^ Department of Physiology, Institute of Biosciences, University of São Paulo, São Paulo, Brazil

**Keywords:** diabetes, gut microbiota, HPA axis, LPS, glucococorticoids

## Abstract

**Introduction:**

Diabetes induces glucocorticoid production in patients and animal models, however, the exact mechanism behind this phenomenon is still elusive. The activation of toll-like receptor (TLR) 4 induces glucocorticoid production by the adrenals. Since diabetic patients showed gut dysbiosis in parallel to an increase in epithelial-intestinal permeability, this study investigates the role of TLR4 activation by gut bacteria-derived lipopolysaccharide on the overproduction of corticosterone in diabetic rodents.

**Methods:**

Diabetes induction was achieved through the intravenous injection of alloxan, followed by treatments with antibiotic therapy or TLR4 antagonist (TAK-242) for 14 consecutive days.

**Results:**

Diabetic animals showed an increase in plasma corticosterone levels as well as overexpression of TLR4 and Toll/IL-1R domain-containing adaptor-inducing IFN-β (TRIF) in the adrenals. Diabetic mice also showed gut dysbiosis, with an increase in the relative proportion of potentially pathogenic bacteria. We observed morphological alterations as well as increased inflammation in the colon with a predominance of a Th17 cytokine profile in diabetic mice, in parallel to an increase in the epithelial-intestinal permeability and lipopolysaccharide content in the adrenals. TAK-242 significantly decreased the overexpression of adrenocorticotropic hormone receptor and 11β-hydroxysteroid dehydrogenase type 1 in the adrenal glands of diabetic mice. Furthermore, both TLR4 antagonist and TLR4 mutant mice (C3H/HeJ) induced a significant reduction in plasma corticosterone levels in diabetic mice.

**Conclusion:**

Our findings revealed that gut dysbiosis participates in the exacerbation of corticosterone production by diabetic animals, suggesting that therapeutic strategies that can normalize gut microbiota in diabetics represent promising therapeutic candidates for the treatment of glucocorticoid-induced comorbidities in diabetes.

## 1 Introduction

Diabetes is a chronic metabolic disease characterized by hyperglycemia caused by a decrease in the synthesis and/or action of insulin. In 2021, the International Diabetes Federation (IDF) estimated that 537 million people were living with diabetes, of which approximately 50% have not yet been diagnosed and, consequently, have uncontrolled blood glucose levels (1). In diabetic patients, poorly controlled glycemia is related to the development of several disabling and costly complications, accompanied by an expense of about 700 billion USD in global healthcare (2). Hyperglycemia and the reduction in the production and/or action of insulin culminate in a deep hormonal imbalance in diabetic patients, including the hyperactivity of the hypothalamus-pituitary-adrenal (HPA) axis (3–5). In diabetic patients, the high circulating levels of glucocorticoids, a stress hormone produced by activation of the HPA axis, are correlated with the promotion of several comorbidities, such as neuropathy, depression, and wound healing deficiency (6–9).

Previously, we and others showed that the exacerbation of adrenal glucocorticoid steroidogenesis in diabetic animals is related to high systemic adrenocorticotropic hormone (ACTH) levels combined with overexpression of the ACTH receptor, MC2R, and the steroid machinery, including acute regulatory protein (StAR) and 11β-hydroxysteroid dehydrogenase type 1 (11βHSD1), in the adrenal glands (10–13). Furthermore, we demonstrated that an imbalance in the pro- and anti-inflammatory pathways in the adrenal glands is crucial to the high circulating glucocorticoid levels during alloxan-induced diabetes in rodents. We showed that an increase in the activation of pro-inflammatory Angiotensin (Ang)-II/Ang-II receptor (AT) subtype AT1 pathway is involved in the exacerbation of glucocorticoid production by the adrenal glands in diabetic mice, which is reversed by the stimulation of the anti-inflammatory AT2 receptor (11). Besides, the reduction in the expression and activation of the anti-inflammatory receptor peroxisome proliferator-activated receptor (PPAR)γ in the adrenal glands of diabetic rats also participates in the increased production of glucocorticoids (12).

It is well known that diabetic patients display dysbiosis in their gut microbiota, with a predominance of pathogenic bacteria and an increase in the permeability of the epithelial-intestinal barrier, which results in high levels of bacterial products, such as lipopolysaccharide (LPS) in the circulation (2, 14). In addition, diabetic patients also show a rise in the systemic levels of endogenous activators of TLR4, including 70 kilodalton heat shock protein (HSP70) and high mobility group box 1 protein (HMGB1) (15). Furthermore, monocytes isolated from the peripheral blood of diabetic patients show overexpression of TLR4 and Toll/IL-1R domain-containing adaptor-inducing IFN-β (TRIF) compared to cells obtained from control subjects (15). In addition, human and murine adrenocortical cells express TLR4 (16), and these cells produce glucocorticoids after stimulation with LPS *in vitro* and *in vivo* (17, 18).

In this study, we evaluated whether TLR4 activation by endogenous or gut bacteria-derived TLR4 agonists contributes to the overproduction of corticosterone in diabetic mice. As corticosterone is produced by adrenals, we performed an evaluation of steroidogenic machinery, TLR4, TRIF, and endogenous activators of TLR4 expression in these glands of diabetic animals. To evaluate the microbiota profile and epithelial-intestinal permeability in our model, we analyzed the microbiome in the feces of Swiss-webster mice, histopathological parameters and cytokine profile in the colon, and quantified the permeabilization of Dextran FITC400 from gut to bloodstream, and the levels of LPS in the plasma. We also included in this evaluation the analysis of LPS content in the adrenals of diabetic mice and mice submitted to antibiotic therapy. To assess the effect of TLR4 on the overproduction of corticosterone by diabetic mice, we treated the mice with TLR4 antagonist TAK-242 and induced diabetes in the TLR4 mutant mice C3H.HeJ. We obtained direct evidence that gut bacteria-derived LPS is involved in the exacerbation of corticosterone production by diabetic animals through the activation of TLR4 in the adrenal glands.

## 2 Material and methods

### 2.1 Chemicals

Alloxan monohydrate, ampicillin, FITC D4000, LPS (Escherichia coli serotype 026: B6), metronidazole, and neomycin were purchased from Sigma Chemical Co. (Saint Louis, MO, USA). Ethanol, methanol, and xylene were purchased from Merck (Rio de Janeiro, RJ, Brazil). Ketamine and xylazine were obtained from Syntec (São Paulo, SP, Brazil), and sodium heparin and sterile saline solution from Roche (São Paulo, SP, Brazil). TAK-242 was purchased from MedChemExpress (Monmouth Junction, NJ, USA). All solutions were prepared immediately before use.

### 2.2 Diabetes induction and stimulation

Male Wistar rats (4–6 weeks old, weighing 200 to 250 g), Swiss-Webster (5–6 weeks old, weighing 20 to 25 g), C3H.He (4–6 weeks old, weighing 18 to 20 g), and C3H.HeJ mice (4–6 weeks old, weighing 18 to 20 g) were obtained from the Institute of Science and Technology in Biomodels (ICTB) from Oswaldo Cruz Foundation (Fiocruz). All procedures used were approved by the Committee on Use of Laboratory Animals of the Oswaldo Cruz Institute (CEUA-IOC/Fiocruz, licenses L-027/2016 and L-004/2024). Animals were housed in groups of up to four in a temperature (22 °C ± 2 °C), humidity, and light-controlled (12 h light/dark period) colony room, with *ad libitum* access to food and water.

Diabetes was induced in 12-hour fasted (water *ad libitum*) animals by a single intravenous injection of alloxan monohydrate (40 and 65 mg/kg in rats and mice, respectively) (19, 20). Non-diabetic animals received an equivalent dose of the vehicle (sterile saline 0.9%). Seven days after the alloxan injection, the blood glycemia was determined with a glucose monitor (Freestyle Optium, Abbott Brasil, Rio de Janeiro, RJ, Brazil) from samples obtained from the tail vein. Only animals showing blood glucose levels greater than 15 mmol/L were considered diabetic and included in the experiment. Twenty-one days after diabetes induction, some rats received a single challenge with LPS (100 ng/cavity, i.p.) and were euthanized after 30 min or 60 min (17). We injected an equivalent dose of the vehicle (sterile saline 0.9%) in control rats. Animal euthanasia was performed with ketamine (140 mg/kg, i.p.) and xylazine (20 mg/kg, i.p.), as previously reported (21), 21 days after diabetes induction.

### 2.3 Hormone quantification

Animals were euthanized during the nadir (08:00h) of the circadian rhythm, as previously described (Ventura et al., 2020). Blood was immediately collected from the abdominal aorta with heparinized saline (40 U/mL), centrifuged for 10 min at 4 °C and 433 × g, and stored at -20°C until use. Plasma corticosterone and insulin were quantified by radioimmunoassay kit following the manufacturer’s guidelines (MP Biomedical, Irvine, CA, USA), using a gamma counter (ICN Isomedic 4/600 HE; ICN Biomedicals Inc., Costa Mesa, USA).

### 2.4 Immunohistochemistry staining

The left adrenal glands, cleaned of surrounding fat, and the colon, cleaned of feces, using sterile PBS, were obtained from animals. Immunohistochemistry in paraffinized adrenal and colon histological sections was performed as previously described (Insuela et al., 2019). The primary antibodies used were specific polyclonal goat anti-TLR4 (1:50; Santa Cruz Biotechnology, Santa Cruz, CA, USA) and specific antibody monoclonal mouse anti-LPS (1:100; Hycult Biotech Inc. Wayne, PA, USA). The secondary antibodies used were horseradish peroxidase-conjugated streptavidin (HRP) (polyclonal anti-goat and monoclonal anti-mouse IgG (1:100), R&D Systems, Minneapolis, MN, USA).

### 2.5 Western blot analysis

The cleaned right adrenal glands were homogenized in RIPA buffer containing protease and phosphatase inhibitor cocktails. After quantifying protein content by the BCA method (D’Almeida et al., 2017), 60 µg total protein/lane was resolved on 14% sodium dodecyl sulfate-polyacrylamide gel electrophoresis, and then electrotransferred to a nitrocellulose membrane using a semi-dry transfer apparatus (Trans-Blot SD; Bio-Rad, Hercules, CA, USA). Next, the immunoblot was performed as previously described (Insuela et al., 2019). Primary antibodies against the following proteins were used: anti-11β-HSD1 (1:200; Santa Cruz Biotechnology), anti-MC2R (1:200; Santa Cruz Biotechnology), anti-StAR (1:250; Santa Cruz Biotechnology), anti-TLR4 (1:250; Santa Cruz Biotechnology), and anti-TRIF (1:200; Thermo Fisher Scientific, Waltham, MA, USA). The housekeeping anti-β-actin (1:1000; Santa Cruz Biotechnology) was used as the standard.

### 2.6 DNA extraction

Approximately 100 mg of mouse feces was used for DNA extraction using the QIAamp DNA Stool Minikit (Qiagen, Germany) with some modifications. Mechanical lysis was performed by transferring the stool to PowerBead Garnet tubes (Qiagen), adding 1 mL of InhibitEX buffer to each sample, and incubating at 95°C for 5 min. Afterward, samples were vortexed at maximum speed for 10 min, centrifuged at 13,000 × g for 1 min, and the supernatant was collected. The next steps were followed according to the manufacturer’s recommendations. DNA was stored at -80°C until analysis.

### 2.7 16S rRNA sequencing

Illumina protocols were used for sequencing 16S rRDNA amplicon libraries. We amplified the variable regions V3-V4 of the 16S rRNA gene using primers 5’- CCTACGGGNGGCWGCAG-3’ (forward) and 5’- GACTACHVGGGTATCTAATCC-3’ (reverse) and the enzyme KAPA HiFi HotStart ReadyMix (Roche, Pleasanton, USA). After preparation, libraries were sequenced on the MiSeq system with chemistry v2–500 cycles. Sequencing was performed at the Plataforma de Sequenciamento de Ácidos Nucleicos de Nova Geração – RPT01J of Fundação Oswaldo Cruz (Rio de Janeiro, Brazil). Sequencing data analysis was performed as previously described (Hardoim et al., 202 3). Initially the sequences were quality checked with FastQC (Wingett and Andrews 2018). Sequence data were quality-filtered and trimmed using Trimmomatic version 0.36 (Bolger et al., 2014), truncating reads if the quality dropped below 20 in a sliding window of 4 bp. Further processing was performed with USEARCH version 11.0.667 (Edgar 2013) where sequences were merged, and the sequencing reads were quality-filtered excluding reads with < 395 or > 470 nucleotides. Additionally, reads with more than one ambiguous base or an expected error of > 1 were also removed from the dataset. Filtered sequences were denoised and clustered into unique sequences (Amplicon Sequence Variants, ASV) using the UNOISE3 algorithm (Edgar 2016a) implemented in USEARCH. Chimeric sequences were removed *de novo* during clustering and subsequently in reference mode using UCHIME2 (Edgar 2016b) with the Genome Taxonomy Database (GTDB, Parks et al., 2020). The ASVs were classified against GTDB using the BLCA algorithm (Gao et al., 2017). Sequences from mitochondria and chloroplasts were removed from the dataset based on the Greengenes 13_5 taxonomy (McDonald et al., 2012). The resulting ASV table contained a total of 488,313 reads, with an average number of reads per sample of 48,831 (ranging between 2,797 to 154,310 reads per sample). The generated ASV table was then processed using the MicrobiomeAnalyst online software suite (https://www.microbiomeanalyst.ca/) in Marker Data Profiling mode. All sequences generated in this study were deposited as a Sequence Read Archive in the NCBI database with Bioproject ID PRJNA1253081 (SAMN48063965- SAMN48063969 for 16S rRNA gene of the control samples and SAMN48063970- SAMN48063974 for 16S rRNA gene for the treatment samples).

### 2.8 Histological analysis

Colons were fixed in Millonig’s buffer solution (pH = 7.4) with 4% paraformaldehyde for 48 h to preserve tissue architecture. Then, 4 µm thick sections were stained with hematoxylin and eosin to measure villi height and crypt depth, with determinations made in 4–10 randomly selected villi and crypts, besides muscular thickness, and inflammation score, using six randomly selected fields. The inflammation score was calculated using the following parameters: inflammatory infiltrate, extent of inflammation, and inflammatory percentage impairment (**Table 1**). Histologic sections were stained with periodic acid–Schiff (PAS) for measuring mucus production and PAS & alcian blue to quantify mucus thickness. Images were scanned using a 3DHISTECH–Pannoramic MIDI whole slide scanner (Budapest, Hungary) and captured with a 20× objective lens. Analyses were performed using ImageJ™ Software (NIH, Bethesda, MD, USA) or Pannoramic Viewer Software (3DHISTECH; Budapest, Hungary).

**Table 1:** Criteria adopted for analyzing the inflammatory score in the colon.

Criteria adopted			
*Inflammation score*	Inflammatory infiltrate	1	None
		2	Discreet
		3	Moderate
		4	Severe
	Extension	1	None
		2	Mucosa
		3	Mucosa and submucosa
		4	Transmural
	Inflammatory percentage impairment	1	1-25%
		2	26-50%
		3	51-75%
		4	76%-100%
Maximum total		12	

### 2.9 Cytokine quantification

We homogenized the colons in cold lysis buffer containing 0.1% Triton X-100 and a Complete protease inhibitor cocktail (F. Hoffmann-La Roche Ltd.) in PBS 1X, and centrifuged at 13,000 × g for 10 min at 4°C. Then, levels of IL-1β, IL-4, IL-6, IL-10, IL-17, IL-22, TGF-β1, and TNF-α from supernatants of colon lysates were measured using commercially available ELISA kits (R&D Systems and Thermo Fisher Scientific). All assays were performed according to the manufacturer’s instructions.

### 2.10 Intestinal permeability analysis

Twenty-one days after diabetes induction, we administered Dextran FITC400 (600 mg/kg, gavage) to 12-hour fasted mice (water ad libitum). After one hour, we obtained the plasma for quantification of Dextran FITC400 that crossed from the gut to the bloodstream by fluorescence (excitation of 485 nm and emission of 528 nm), using a plate reader (SpectraMax M5, Molecular Devices, San Jose, CA, USA).

### 2.11 Treatments

Mice were treated with the TLR4 antagonist TAK-242 (3 mg/kg, i.p.) or an antibiotic cocktail containing ampicillin, metronidazole, and neomycin (1 g/L, drinking water) daily, for 14 consecutive days, starting 7 days after diabetes induction. Untreated mice received an equal volume of vehicles (0.1% DMSO, i.p., and drinking water, respectively). Drinking water with or without antibiotic cocktail was changed at least every 3 days. All analyses were performed 24h after the last treatment.

### 2.12 Statistical analysis

The results were expressed as the mean ± standard error of the mean (SEM). All data were evaluated to ensure normal distribution and statistically analyzed by one-way analysis of variance (ANOVA), followed by the Student Newman-Keuls multiple comparison post-test. We used the unpaired student’s t-test when only two experimental groups were compared. All statistical analyses were performed with GraphPad Prism 8 software (La Jolla, CA, USA). Probability values (*P*) of 0.05 or less were considered significant. Statistical analysis of microbiome data was conducted using the MicrobiomeAnalyst software. Alpha diversity (Chao1) was compared using Welch’s t-test. Beta diversity was analyzed at the ASV level, by Principal Coordinate Analysis (PCoA) using the Bray-Curtis index as the distance method and PERMANOVA. Dendrograms were generated at the ASV level, also using the Bray-Curtis index as the distance method and the Ward clustering algorithm. Linear Discriminant Analysis Effect Size (LEfSe) was performed at the genus level, using a *P* value cut-off of 0.05 and an LDA score of 2.0.

## 3 Results

### 3.1 Diabetes induces hypercortisolism and an increase in the expression of functional TLR4 and TRIF in the adrenal glands

**Figure 1:**
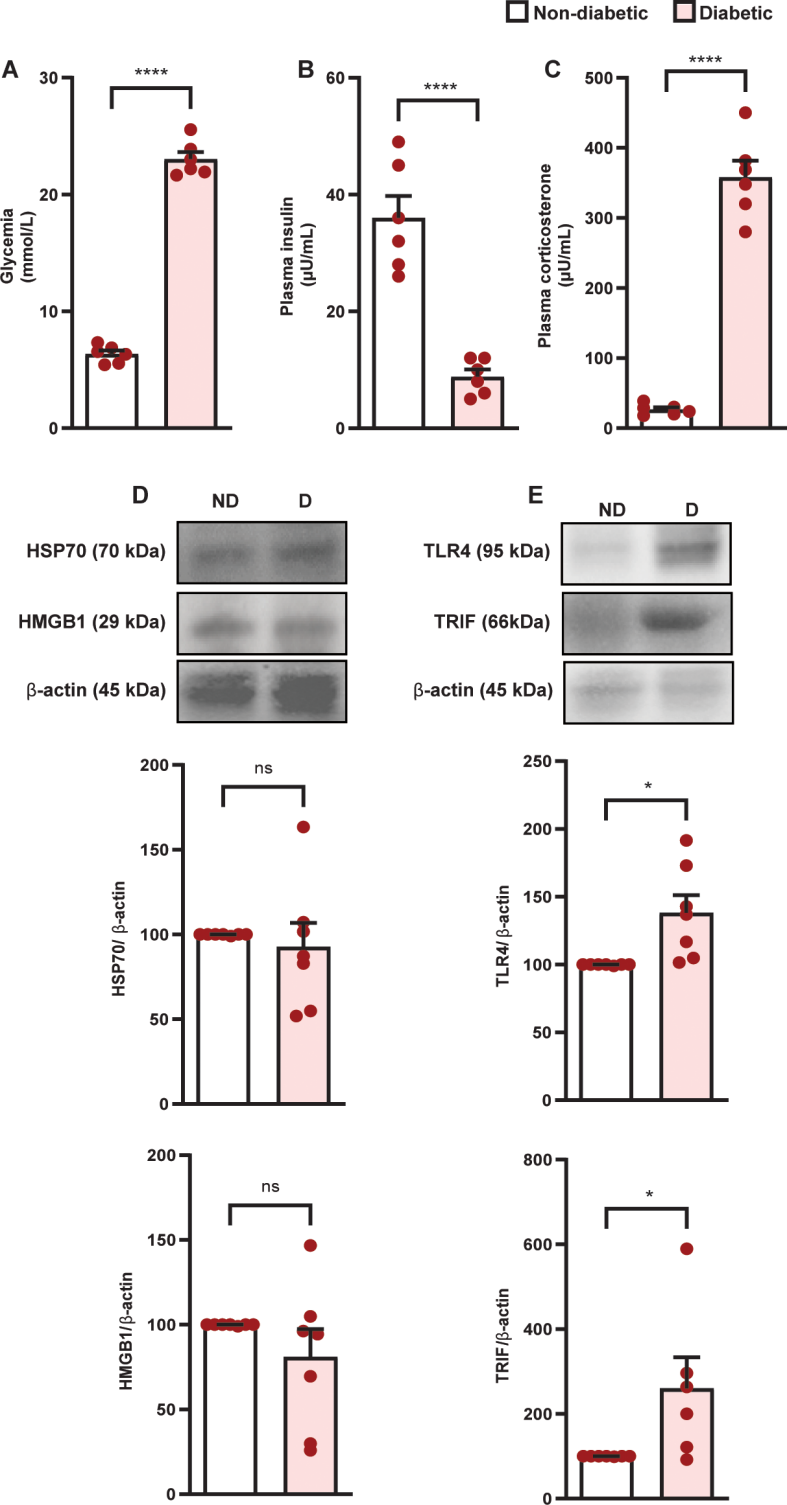
Diabetes induces hypercortisolism in parallel to an increase in the expression of TLR4 and TRIF but does not alter the amount of HMGB1 and HSP70 in the adrenal glands of Swiss-Webster mice. Analyses were performed 21 days after diabetes induction. **(A)** Blood glucose quantification. **(B, C)** Plasma quantification of insulin and corticosterone, respectively, by radioimmunoassay. **(D)** Expression of HMGB1 and HSP70 in the adrenal glands. **(E)** Expression of TLR4 and TRIF in the adrenal glands. Protein expression was determined by western blot. Data were normalized to β-actin and presented as the ratio between target protein levels relative to controls. Each bar represents the mean ± standard error of the mean. Pink dots represent the number of animals analyzed. Statistical analyses were performed by the students’ t-test. ^*^p<0.05. ^****^p<0.0001. ns, non-significant. HMGB1, High mobility group box 1 protein; HSP70, 70 kilodalton heat shock proteins; TLR4, toll-like receptor 4; TRIF, TIR-domain-containing adapter-inducing interferon-β.

**Figure 2:**
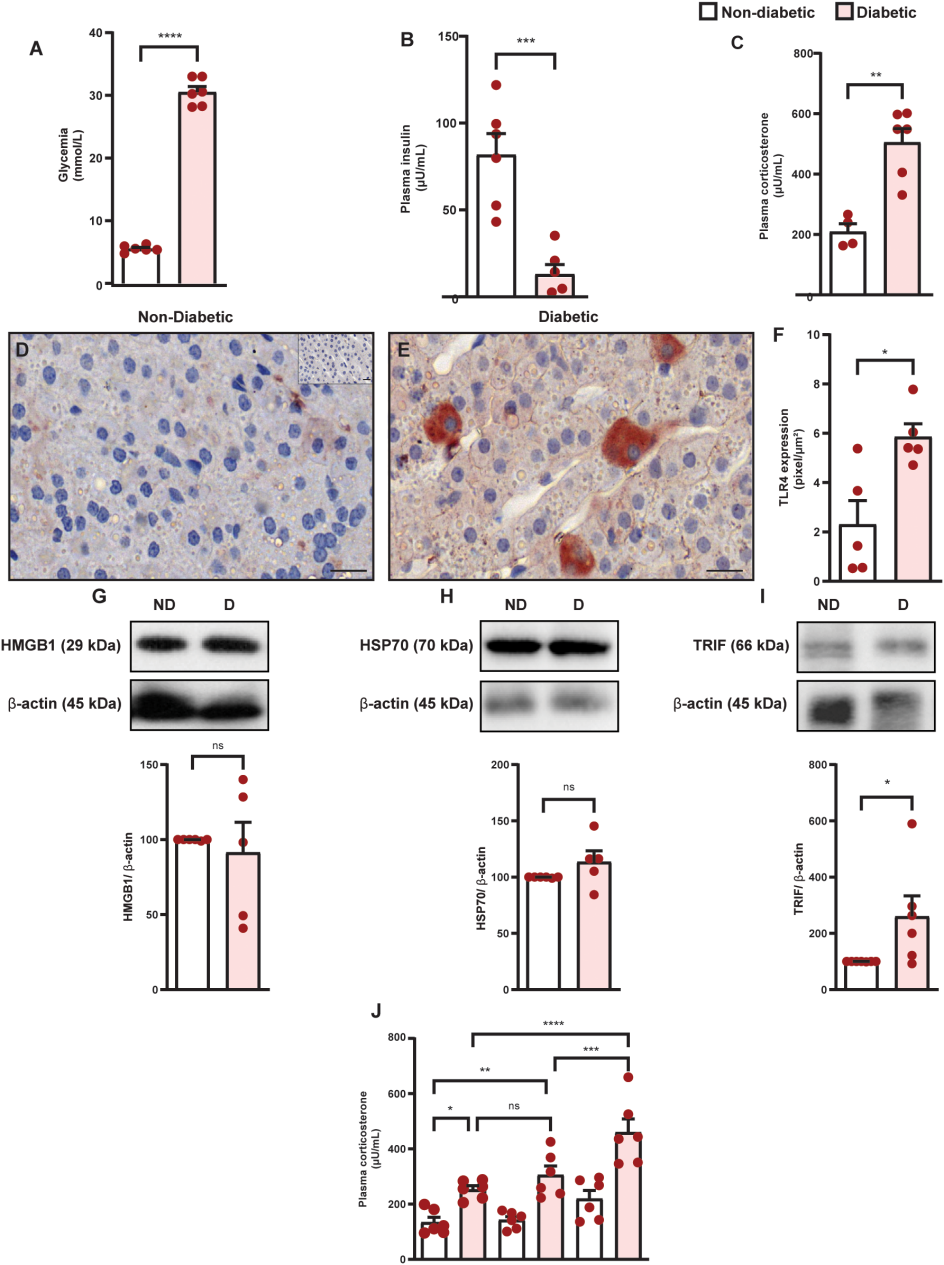
Diabetes induces hypercortisolism in parallel to an increase in the expression of TLR4 and TRIF but does not alter the amount of HMGB1 and HSP70 in the adrenal glands of Wistar rats. Analyses were performed 21 days after diabetes induction. **(A)** Blood glucose quantification. **(B, C)** Plasma quantification of insulin and corticosterone levels, respectively, by radioimmunoassay. Panels show representative photomicrographs of the expression of TLR4 in the zona fasciculate of the adrenal of non-diabetic **(D)** and diabetic rats **(E)** Inserts represent negative controls. **(F)** Quantification of pixels associated with positive TLR4 expression. **(G–I)** HMGB1, HSP70, and TRIF expression in the adrenal glands, respectively, were determined by western blot. Data were normalized to β-actin and presented as the ratio between target protein levels relative to controls. **(J)** Evaluation of LPS-induced plasma corticosterone levels in diabetic rats. Analysis was performed 30 and 60 min after LPS injection (100 ng/mL, i.p.). Each bar represents the mean ± standard error of the mean. Pink dots represent the number of animals analyzed. Statistical analyses were performed by the students’ t-test. In analyzing three or more experimental groups, we used one-way ANOVA followed by Newman–Keuls test. ^*^p<0.05. ^**^p<0.005. ^***^p<0.0005. ^****^p<0.0001. ns, non-significant; Scale bar = 20 μm; HMGB1, High mobility group box 1 protein; HSP70, 70 kilodalton heat shock proteins; LPS, lipopolysaccharide; TLR4, toll-like receptor 4; TRIF, TIR-domain-containing adapter-inducing interferon-β.

Diabetes induced hyperglycemia (**Figure 1A**) and reduction in circulating insulin levels (**Figure 1B**), in parallel to an increase in plasma corticosterone levels (**Figure 1C**) in Swiss-Webster mice, compared to non-diabetic mice. Diabetes did not alter the expression of HMGB1 and HSP70 in the adrenal glands of Swiss-Webster mice (**Figure 1D**, **Supplementary Figure S1A**, **Supplementary Figure S1B** and **Supplementary Figure S1C**); however, it increased the expression of TLR4 and TRIF (**Figure 1E**, **Supplementary Figure S1D**, **Supplementary Figures S1E, F**) compared to non-diabetic mice. We also showed that diabetes induced the same results in Wistar rats (**Figures 2A–I** and **Supplementary Figures S2A–F**). To evaluate whether the increase in the TLR4 signaling machinery in the adrenal glands of diabetic rats could be related to a higher activity of the TLR4 pathway, we challenged rats with LPS, an activator of TLR4, and showed that LPS increased plasma corticosterone levels in diabetic rats 60 min after the challenge, without altering this output in non-diabetic rats (**Figure 2J**).

### 3.2 Diabetes alters gut microbiota profiles of Swiss-Webster mice

**Figure 3:**
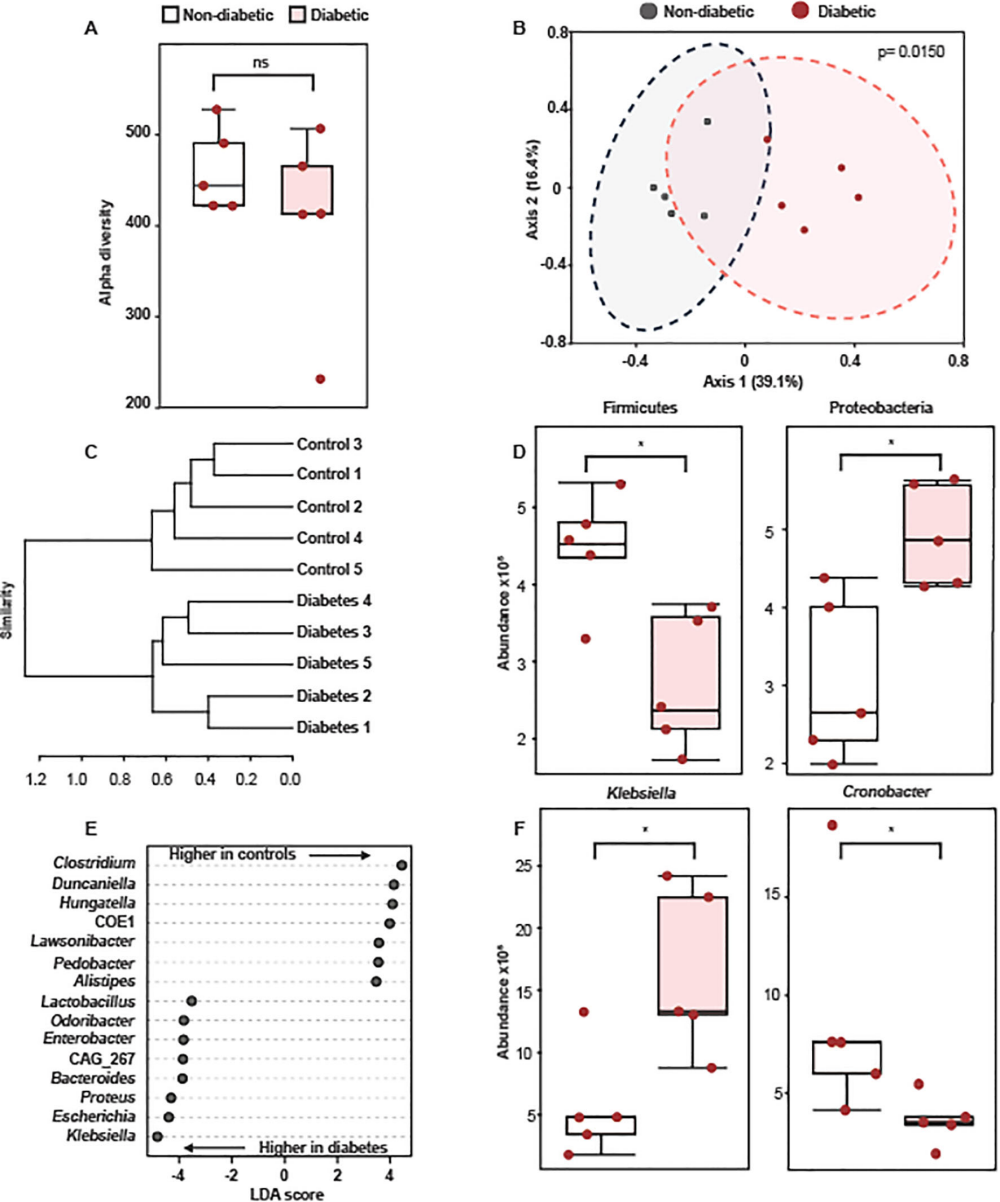
Diabetes alters colonic microbiota composition in Swiss-Webster mice. Analyses were performed 21 days after diabetes induction. **(A)** Alpha diversity of gut microbial communities from non-diabetic and diabetic mice. Statistical analyses were performed by Welch’s t-test. **(B)** Principal Coordinate analysis (PCoA) of fecal bacteriome composition of non-diabetic and diabetic mice. Statistical analyses were performed by Bray-Curtis index as the distance method and PERMANOVA. **(C)** Dendrogram showing the separation of non-diabetic and diabetic mice based on changes in bacteriome composition. Statistical analyses were performed by Bray-Curtis index as the distance method and the Ward clustering algorithm. **(D)** Relative abundance of Firmicutes and Proteobacteria in fecal samples of non-diabetic and diabetic mice. **(E)** LDA scores for the 15 taxa with the highest discriminatory power to differentiate non-diabetic and diabetic mice. **(F)** Relative abundance of *Klebsiella* and *Clostridium* in fecal samples of non-diabetic and diabetic mice. Each bar represents the mean ± standard error of the mean. Pink dots represent the number of animals analyzed. ^*^p<0.05. ns, non-significant.

Since we did not observe an alteration in the expression of HMGB1 and HSP70, which are damage-associated molecular patterns (DAMPs) that activate TLR4, in the adrenal glands of diabetic rats and mice, we evaluated if diabetes changes the gut bacterial composition in Swiss-Webster mice. We did not observe changes in alpha diversity between the bacteriome of non-diabetic and diabetic mice (**Figure 3A**). Nevertheless, we found differences in beta diversity, as attested by the PCoA plot in **Figure 3B** and the dendrogram in **Figure 3C**. Interestingly, diabetes induction significantly changed the relative abundances of some phyla compared to non-diabetic mice, including a decrease in the Firmicutes and an increase in Proteobacteria (**Figure 3D**). We did not observe changes in the Bacteroidetes (data not shown). Furthermore, diabetic mice showed differences in the relative abundances of opportunistic pathogenic bacteria, including an increase in *Klebsiella* and a reduction in *Clostridium* (**Figures 3E, F**).

### 3.3 Diabetes promotes altered crypt-villus morphology and induces inflammation and mucus production in the murine colon

**Figure 4:**
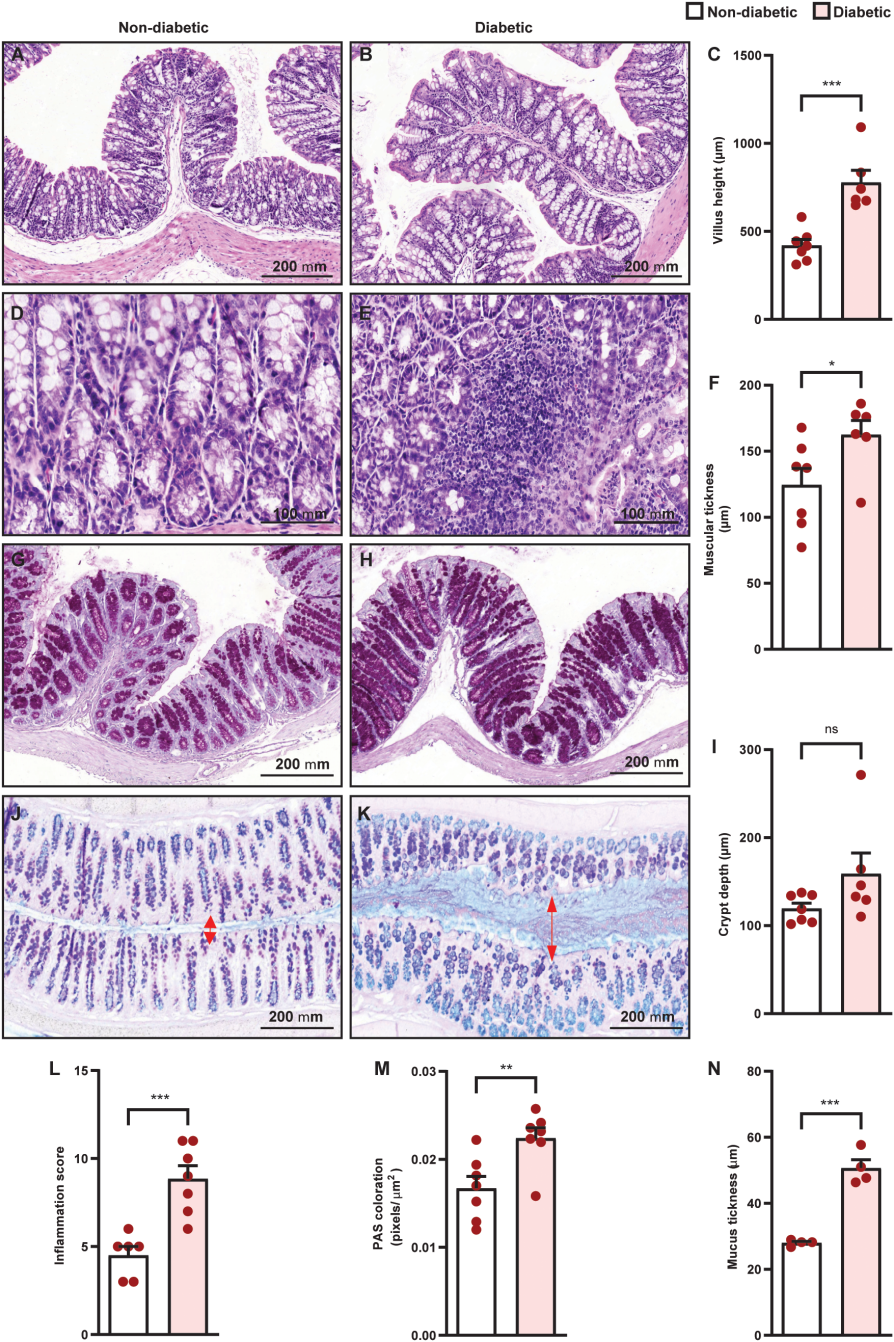
Diabetes induces histopathological alterations in the colon of Swiss-Webster mice. Analyses were performed 21 days after diabetes induction. Panels show representative photomicrographs of colon slices of non-diabetic and diabetic mice stained with hematoxylin and eosin [**(A, B, D, E)**, respectively], PAS [**(G, H)**, respectively], and PAS plus alcian blue [**(J, K)**, respectively]. **(C, F, I, L)** Evaluation of villus height, muscular thickness, crypt depth, and inflammation score, respectively, in colon sections stained with H&E. **(M, N)** Quantification of pixels associated with mucus and mucus thickness in colon sections stained with PAS and PAS plus alcian blue, respectively. Each bar represents the mean ± standard error of the mean. Pink dots represent the number of animals analyzed. Statistical analyses were performed by the students’ t-test. ^*^p<0.05. ^**^p<0.005. ^***^p<0.0005. ns, non-significant. Red arrows indicated the mucus area in the colon sections stained with PAS plus alcian blue. Scale bar = 100 µm **(B, G)** and 200 µm **(A, C, D, F, H, I)**. PAS, periodic acid-Schiff.

**Figure 5:**
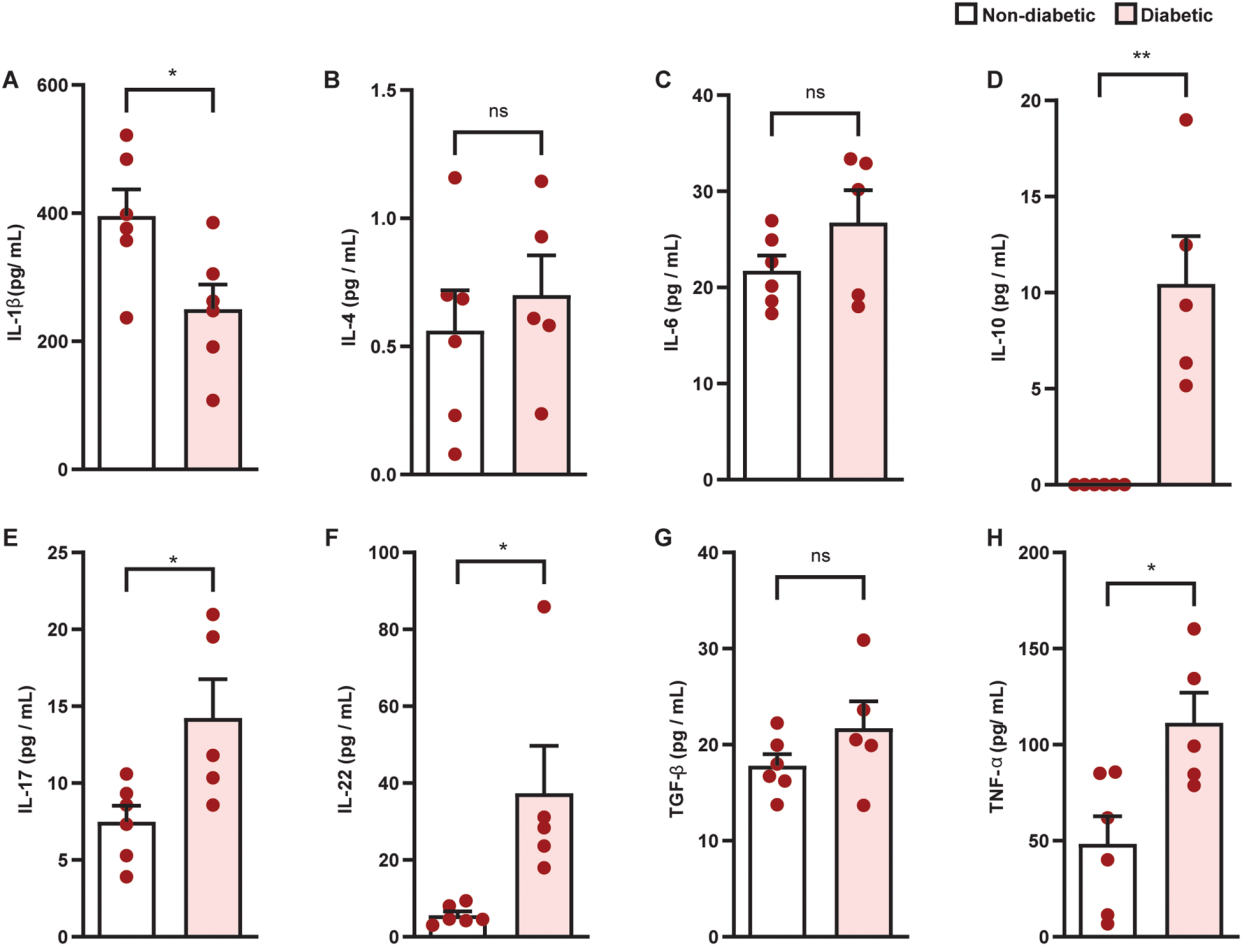
Diabetes alters the production of cytokines in the colon of Swiss-Webster mice. Colon levels of IL-1β **(A)**, IL-4 **(B)**, IL-6 **(C)**, IL-10 **(D)**, IL-17 **(E)**, IL-22 **(F)**, TNF-α **(G)**, and TGF-β1 **(H)** were evaluated 21 days after diabetes induction. Cytokine levels were quantified by ELISA. Each bar represents the mean ± standard error of the mean. Pink dots represent the number of animals analyzed. Statistical analyses were performed by the students’ t-test. ^*^p<0.05. ^**^p<0.005. ns, non-significant; IL, interleukin; TGF, transforming growth factor; TNF, tumor necrosis factor.

Diabetes promoted an increase in villus height (**Figures 4A–C**) and muscular thickness (**Figure 4F**), but did not change crypt depth (**Figure 4I**), in the colon of diabetic mice, compared to non-diabetic controls. Furthermore, diabetes induced inflammatory infiltration (**Figures 4D, E, L**) in the colon, in addition to an increase in mucus production (**Figure 4G, H, M**) and mucus layer thickness (**Figures 4J, K, N**). Diabetes increased IL-10, IL-17, IL-22, and TNF-α levels (**Figures 5D–F, H**), and reduced IL-1β (**Figure 5A**) levels in the colon of diabetic mice compared to non-diabetic controls, without altering the content of IL-4, IL-6, and TGF- β1 (**Figures 5B, C, G**).

### 3.4 Diabetes increases the permeability of the epithelial-intestinal barrier, resulting in a greater influx of LPS to the adrenal glands

**Figure 6:**
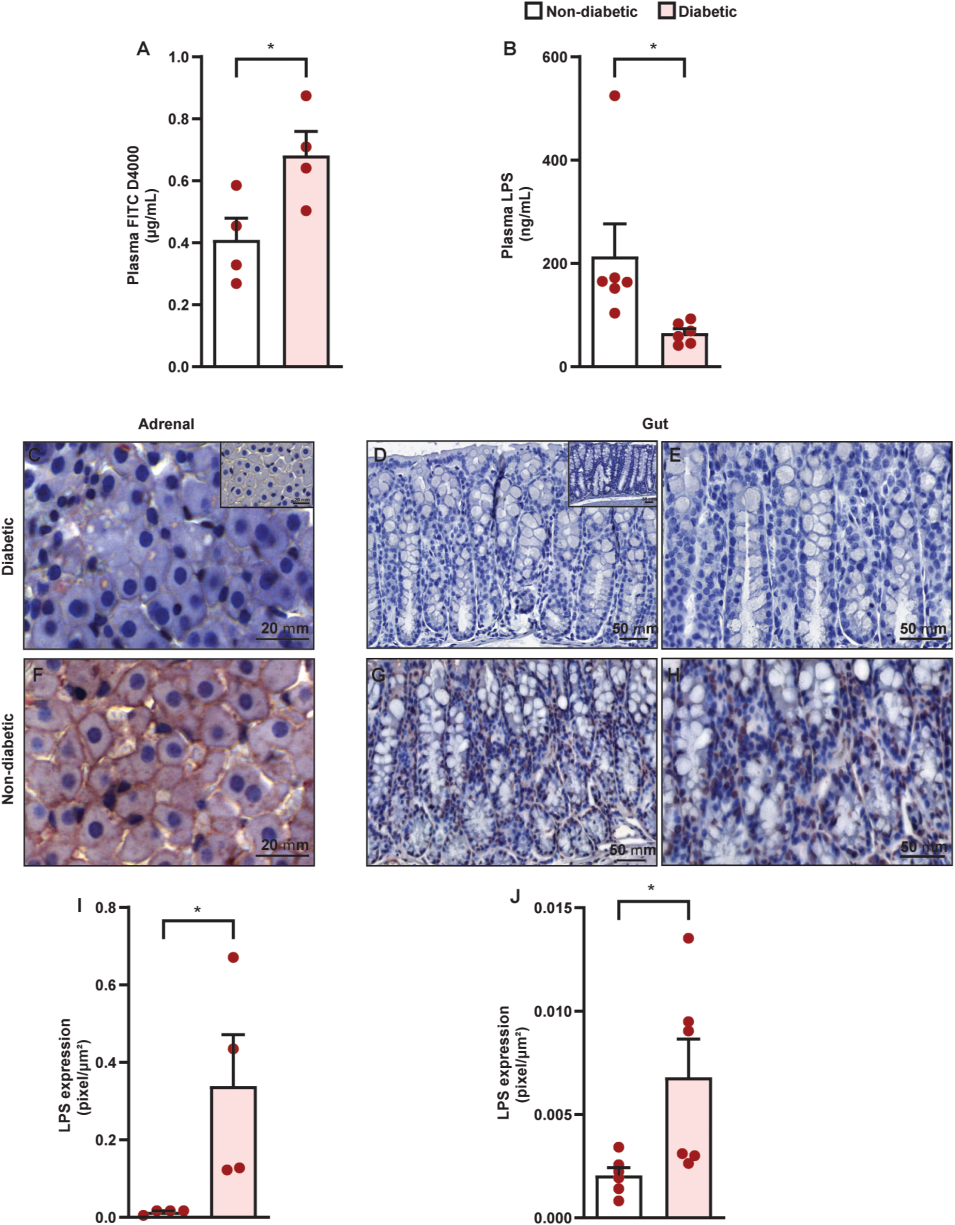
Diabetes induces an increase in epithelial-intestinal permeability in parallel to an influx of LPS into the adrenal glands of Swiss-Webster mice. Analyses were performed 21 days after diabetes induction. **(A)** Quantification of serum levels of FITC D4000–1 h after oral administration by gavage. **(B)** Evaluation of plasmatic levels of LPS by ELISA. Panels show representative photomicrographs of LPS levels in the zona fasciculate of adrenal **(C, F)** and colon **(D, E, G, H)** of non-diabetic **(C–E)** and diabetic mice **(F–H)**. Inserts represent negative controls. **(I, J)** Quantification of pixels associated with LPS levels in adrenals and gut, respectively. Each bar represents the mean ± standard error of the mean. Pink dots represent the number of animals analyzed. Statistical analyses were performed by the students’ t-test. ^**^p<0.05. Scale bar = 20 μm **(C, F)**, 50 µm **(D, G)**, and 100 µm **(E, H)**. FITC, fluorescein isothiocyanate; LPS, lipopolysaccharide.

We observed that diabetic mice presented an increase in the influx of FITC D4000 from the intestinal lumen to blood compared to non-diabetic mice (**Figure 6A**), showing a reduction in the epithelial-intestinal barrier. However, diabetic mice presented a reduction in plasma LPS levels compared to non-diabetic mice (**Figure 6B**). We also showed an increase in the levels of LPS in both the adrenals (**Figures 6C, F, I**) and colon (**Figures 6D, E, G, H, J**) of diabetic mice compared to non-diabetic controls.

### 3.5 Antibiotic therapy reduces plasma corticosterone levels and the overexpression of TLR4 and TRIF in the adrenals of diabetic mice

**Figure 7:**
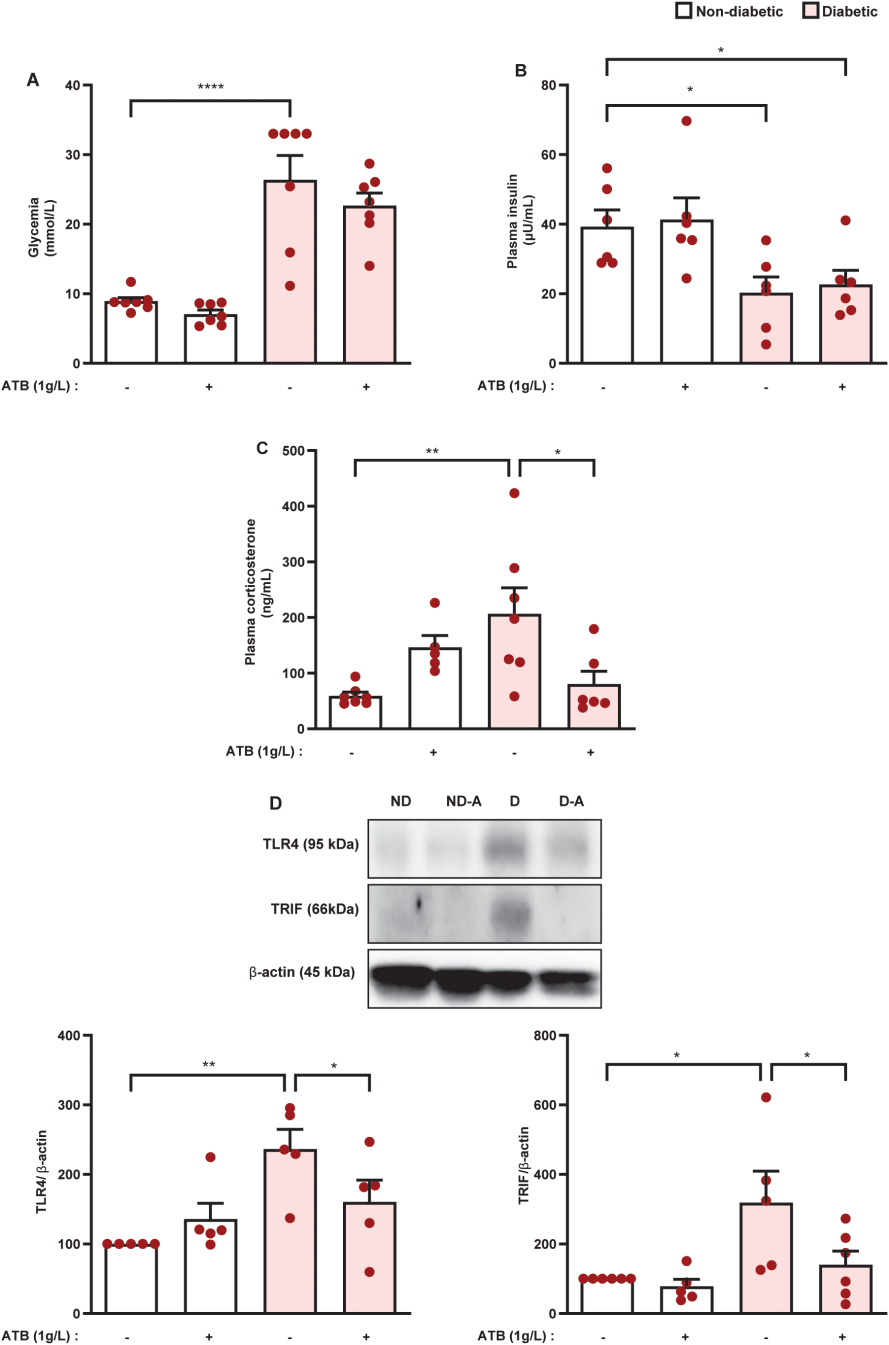
Antibiotic therapy decreases plasma corticosterone levels in parallel to a reduction in TLR4 and TRIF expression in the adrenal glands of diabetic mice. Seven days after diabetes induction, mice were treated with an antibiotic cocktail (metronidazole, neomycin, and ampicillin, 1g/L, drinking water), for 14 consecutive days. Untreated animals receive an equal amount of vehicle (drinking water). Analyses were performed 21 days after diabetes induction. **(A)** Blood glucose quantification. **(B, C)** Plasma quantification of insulin and corticosterone, respectively, by ELISA. **(D)** Expression of TLR4 and TRIF in the adrenal glands of non-diabetic and diabetic mice performed by western blot. Data were normalized to β-actin and represented as the ratio between target protein levels relative to controls. Each bar represents the mean ± standard error of the mean. Pink dots represent the number of animals analyzed. Statistical analyses were performed by one-way ANOVA followed by Newman–Keuls test. ^*^p<0.05. ^**^p<0.005. ^****^p<0.0001. ATB, antibiotic cocktail; TLR4, toll-like receptor 4; TRIF, TIR-domain-containing adapter-inducing interferon-β.

The increase in permeability of the epithelial intestinal barrier, the enrichment of potentially pathogenic bacteria in the gut microbiota, and the increase in LPS levels in the adrenal glands of diabetic mice suggest that endotoxins of pathogenic bacteria present in the intestinal microbiota of diabetic mice are involved in the hypercortisolism noted in those animals. To test this hypothesis, we treated animals with an antibiotic cocktail containing ampicillin, metronidazole, and neomycin. Antibiotic therapy significantly reduced blood glucose levels in diabetic mice compared with untreated diabetic mice, although antibiotic-treated animals remained hyperglycemic. Nevertheless, antibiotic therapy did not alter blood glucose levels in non-diabetic mice (**Figure 7A**). Even though antibiotic therapy decreased blood glucose levels in diabetic mice, it did not alter plasma insulin levels in either non-diabetic or diabetic mice (**Figure 7B**). Antibiotic therapy also significantly reduced plasma corticosterone levels in diabetic mice (**Figure 7C**). Lastly, we showed that antibiotic therapy reduced the expression of TLR4 and TRIF in the adrenal glands of diabetic mice (**Figure 7D** and **Supplementary Figures S3A–C**), without modifying these outputs in non-diabetic animals.

### 3.6 TLR4 blockage restores corticosterone levels in diabetic mice through decreased steroidogenesis

**Figure 8:**
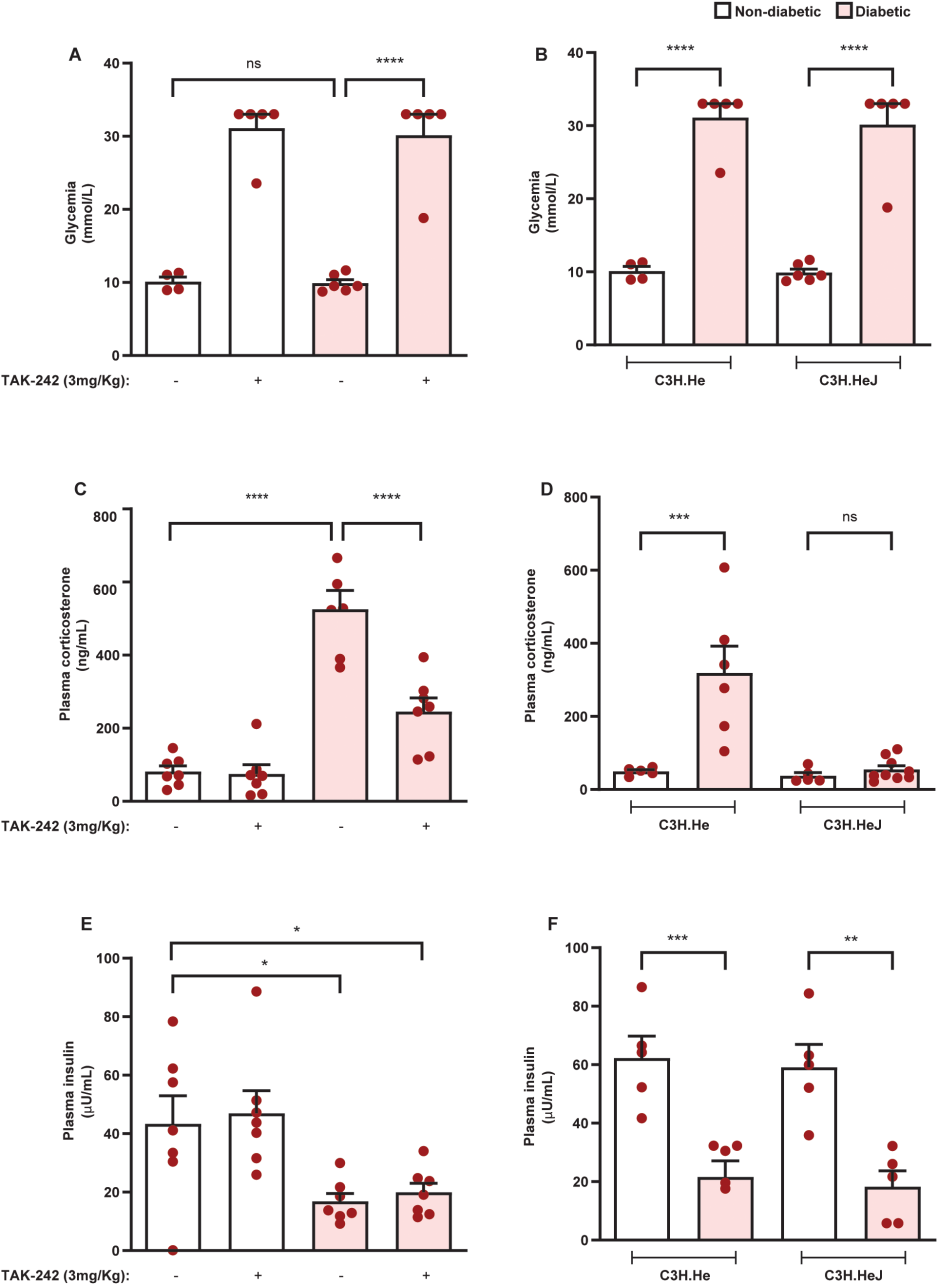
Blockade of TLR4 inhibits hypercortisolism in diabetic mice. Seven days after diabetes induction in Swiss-Webster mice, animals were treated with the TLR4 antagonist TAK-242 (3 mg/kg, i.p.) once a day for 14 consecutive days. Control animals receive an equal amount of vehicle (0.1% DMSO). Analyses were performed 21 days after diabetes induction. **(A, B)** Blood glucose quantification in diabetic mice treated with TAK-242 and animals with a mutation in TLR4, respectively. **(C, D)** Plasma quantification of insulin levels in diabetic mice treated with TAK-242 and TLR mutant animals, respectively. **(E, F)** Plasma quantification of corticosterone levels in diabetic mice treated with TAK-242 and TLR4 mutant animals, respectively. Insulin and corticosterone levels were measured by ELISA. Each bar represents the mean ± standard error of the mean. Pink dots represent the number of animals analyzed. Statistical analyses were performed by one-way ANOVA followed by Newman–Keuls test. ^*^p<0.05. ^**^p<0.005. ^***^p<0.0005. ^****^p<0.0001. ns, non-significant; TAK, TAK-242.

Since antibiotic therapy reduced the hypercortisolism of diabetic mice as well as the expression of TLR4 and TRIF in the adrenals, we evaluated the role of the TLR4 pathway in the exacerbation of adrenal steroidogenesis in diabetic mice. To do this, we used pharmacological and genetic approaches. Treatment with the TLR4 antagonist TAK-242 did not alter blood glycemia and plasma insulin levels in non-diabetic and diabetic mice (**Figures 8A, C**, respectively). Furthermore, TLR4 mutant mice displayed hyperglycemia and hypoinsulinemia after diabetes induction to the same extent as control mice (**Figures 8B, D**, respectively). Nevertheless, both TLR4 antagonist treatment and TLR4 mutation in diabetic animals resulted in reduced plasma corticosterone levels compared to untreated and wild-type diabetic mice (**Figures 8E, F**, respectively). In addition, neither approach to block TLR4 activation modified plasma corticosterone levels in non-diabetic mice.

**Figure 9:**
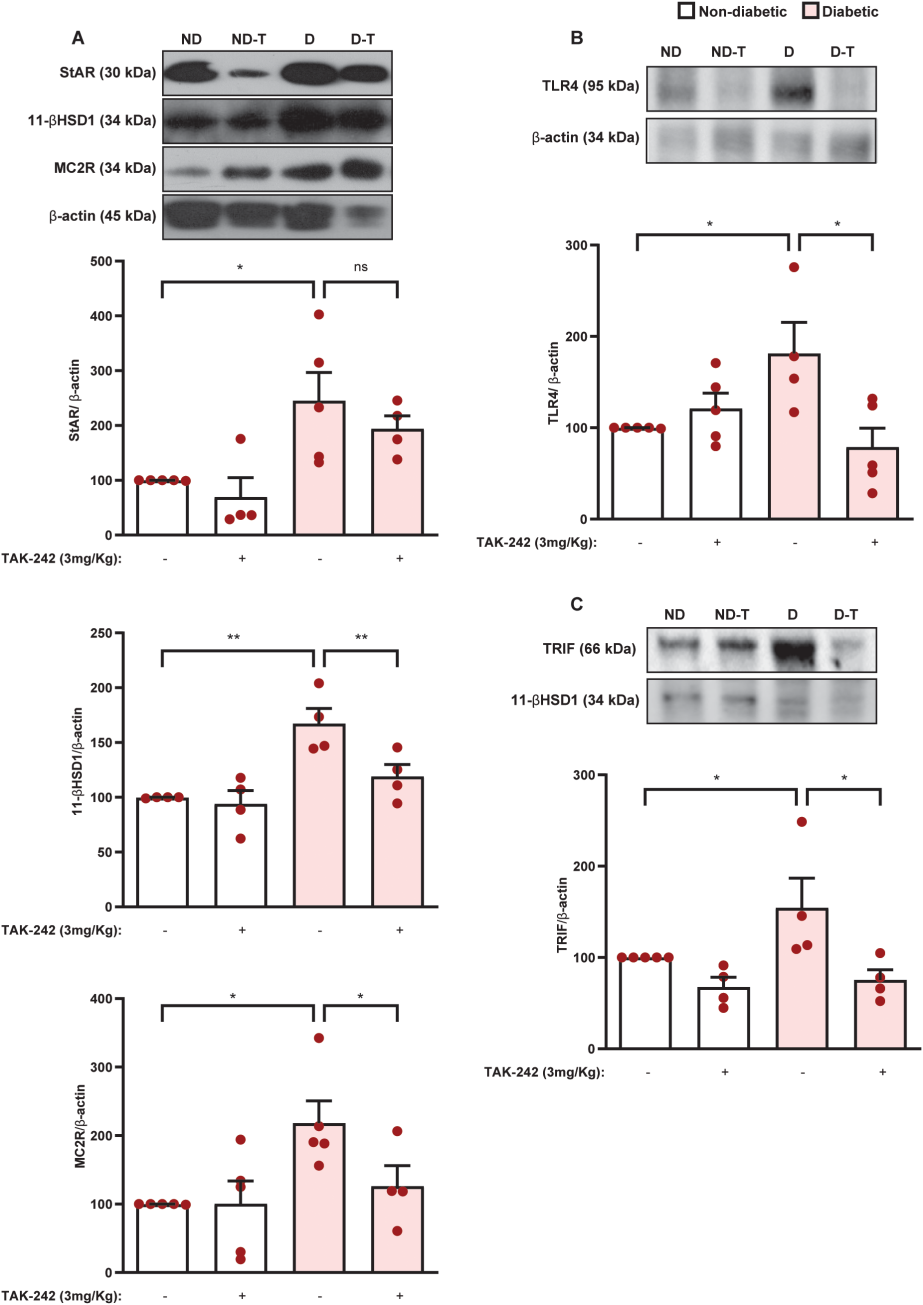
Suppression of TLR4 signaling by TAK-242 represses expression of the steroidogenic machinery, TLR4, and TRIF in the adrenal glands of diabetic mice. Seven days after diabetes induction, animals were treated with the TLR4 antagonist TAK-242 (3 mg/kg, i.p.) once a day for 14 consecutive days. Control animals received an equal amount of vehicle (0.1% DMSO). Analyses were performed 21 days after diabetes induction. **(A)** Expression of StAR, 11βHSD1, and MC2R in the adrenal glands. Expression of TLR4 **(B)** and TRIF **(C)** in the adrenal glands. Protein expression was determined by western blot. Data were normalized to β-actin and represented as ratios between target protein levels relative to controls. Each bar represents the mean ± standard error of the mean. Pink dots represent the number of animals analyzed. Statistical analyses were performed by one-way ANOVA followed by Newman–Keuls test. ^*^p<0.05. ^**^p<0.005. ns, non-significant; 11βHSD1, 11β-Hydroxysteroid dehydrogenase type 1; MC2R, melanocortin receptor 2; StAR, steroidogenic acute regulatory protein; TAK, TAK-242; TLR4, toll-like receptor 4; TRIF, TIR-domain-containing adapter-inducing interferon-β.

Then, we evaluated the effect of TAK-242 on the expression of the steroidogenic machinery in the adrenal glands of diabetic mice. We showed that the overexpression of 11βHSD1 and MC2R noted in the adrenals of diabetic mice was sensitive to treatment with TAK-242, although the expression of StAR was unaffected (**Figure 9A** and **Supplementary Figures S4A–D**). Treatment with TAK-242 did not alter these outputs in the adrenal glands of non-diabetic mice. Furthermore, treatment with TLR4 antagonist downregulated the expression of both TLR4 (**Figure 9B**, **Supplementary Figures S4E, F**) and TRIF (**Figure 9C**, **Supplementary Figures S4G, H**) in the adrenal glands of diabetic mice compared to untreated controls, without modifying these outputs in non-diabetic mice.

## 4 Discussion

This study provides new viewpoints on the involvement of gut-adrenal interactions on the exacerbation of adrenal glucocorticoid steroidogenesis in diabetes. We showed that both diabetic mice and rats displayed overexpression of TLR4 and TRIF in the adrenal glands, an increase in the diversity of pathogenic bacteria and inflammation in the gut, increased epithelial-intestinal barrier permeability, and elevated levels of LPS in the adrenal glands, without altering the content of HMGB1 and HSP70 in this organ. Antibiotic therapy decreased plasma corticosterone levels in diabetic mice in parallel to a down-regulation of TLR4 and TRIF expression in the adrenal glands. In addition, both mutation of TLR4 and treatment with a TLR4 antagonist inhibited the hypercortisolism observed in diabetic mice. The TLR4 antagonist-induced reduction of adrenal glucocorticoid steroidogenesis observed in diabetic mice was related to a decrease of TLR4, TRIF, MC2R, and 11βHSD1 expression in the adrenal glands. Our findings indicate that the activation of the TLR4 pathway induced by endotoxins from pathogenic bacteria found in the gut microbiota of diabetic mice could account for the exacerbation of corticosterone production by the adrenal glands.

Alloxan induces diabetes through the generation of reactive oxygen species (ROS) that mediate variable β-cell toxicity. In some cases, alloxan can destroy all β-cell, exhausting blood insulin levels and culminating in the death of animals. Nevertheless, in general, alloxan induces hyperglycemia stably over time (22, 23). In this study, we did not see any death after alloxan injection in rats and mice, probably because we analyzed them in a relatively short time after administration of the diabetogenic agent. Although we did not follow glycemia over time in this work, we previously showed quite similar levels of blood glucose 24h, 48h, and 72h after alloxan injection in Wistar rats compared to values obtained in this work. Furthermore, in all animal strains evaluated, we showed the same increase in the blood glucose levels and a similar decrease in plasma insulin levels, suggesting glycemic stability in our model of diabetes independent of used rats or different strains of mice (12). However, our model of diabetes has some limitations, including β-cell damage by a mechanism without the participation of an autoimmune reaction and the fact that alloxan directly induces liver and kidney damage (24).

It is well known that LPS induces glucocorticoid production by adrenocortical cells *in vitro* and *in vivo* (17, 18) and that diabetic patients display an increase in circulating levels of HSP70 and HMGB1 (15), which are endogenous activators of TLR4. However, the involvement of LPS in the exacerbation of adrenal glucocorticoid steroidogenesis in diabetic animals remained elusive. First, we showed that both diabetic rats and mice overexpress TLR4 and TRIF in the adrenal glands, while levels of HSP70 and HMGB1 remain unaltered. Furthermore, we noted that LPS induced an increase in plasma corticosterone levels in diabetic rats 60 min after the challenge, without altering circulating levels of this hormone in non-diabetic controls. These data suggest that the activation of the TLR4-TRIF pathway can be involved in the hypercortisolism of diabetic animals; however, the participation of DAMP activators of TLR4 in this phenomenon remained elusive. In this work we used Wistar rats and Swiss-Webster mice due to their genetic variability, making them more representative of natural populations and human diversity. By comparing these species with different genetic backgrounds, we minimize bias from species-specific traits. Immune responses in these two outbred strains are quite similar, with comparable leukocyte counts in the blood, however, no direct comparison of their gut microbiota is available, mice microbiota seem closer to humans than that of rats (25).

We hypothesized that bacterial products from the gut microbiota could participate in the exacerbation of corticosterone production by the adrenal glands of diabetic animals. To test this hypothesis, we evaluated the gut bacteriome content of diabetic mice. We showed that diabetic mice displayed marked changes in gut bacteriome composition, with a reduction in the relative abundance of the Firmicutes, which is composed predominantly of commensal bacteria, and an increase in the Proteobacteria, known to harbor various human pathogens. Among the pathogenic bacteria present at higher levels in the gut bacteriome of diabetic mice, *Escherichia coli*, *Klebsiella pneumoniae*, *Klebsiella variicola*, and *Proteus mirabilis* stand out. However, it is important to note that sequencing of regions V3-V4 of the 16S rRNA gene often does not allow confident taxa identification at the species level. Therefore, these species assignments need validation before more solid conclusions can be drawn. Nevertheless, this gut dysbiosis noted in diabetic mice agrees with what is observed in diabetic patients with uncontrolled glycemia (2, 14). Our data suggests that pathogenic bacteria found in the gut bacteriome of diabetic mice can be the source of TLR4 activators, including LPS, that are increased in the adrenal glands of these animals.

It is well known that gut dysbiosis is usually accompanied by structural changes in the colon, including local inflammation, followed by increased permeability of the epithelial-intestinal barrier (26). We showed that diabetic mice displayed increased villus height and muscular thickness in the colon, which can be an indication of increased intestinal epithelium and muscle cell proliferation, affecting the absorption of nutrients and resulting in unhealthy intestinal conditions (27). Furthermore, we noted the development of an inflammatory response, indicated by increased mucus production in the colon of diabetic mice. Although in commensal homeostasis the mucus layer protects the tissue against microbial penetration (28), in mouse models of colitis and in patients with ulcerative colitis, bacteria penetrate the mucus and are found close to the non-inflamed epithelium (29), indicating a defect in mucus barrier quality. Therefore, high mucus production in the colon of diabetic mice is not necessarily associated with greater epithelial-intestinal barrier integrity. Since the precise balance of immune surveillance and tolerance is crucial to maintaining epithelial-intestinal barrier integrity and uncontrolled immune responses caused by gut microbiota disturbances can break this barrier (30), we evaluated the profiles of pro- and anti-inflammatory cytokines in the gut of diabetic mice. We showed that diabetic mice displayed an increase in IL-10, IL-17, IL-22, and TNF-α levels and a decrease in IL-1β content in the colon compared to non-diabetic mice, without altering the levels of IL-4, IL-6, and TGF-β1. Our data indicate that diabetes leads to a predominantly Th17-type immune response profile in the colon, due to increased levels of IL-17, IL-22, and TNF-α. Although the overproduction of Th17 cytokines in the gut was previously shown in non-obese diabetic (NOD) mice, this phenomenon was demonstrated only in the pre-diabetic period (31, 32). Our work was the first to show a Th17 profile in the gut of type 1 diabetic mice. This Th17 signature in the gut, with an increase in Th17 cells in the lamina propria, is involved in the progression of intestinal inflammation and, consequently, in the break of the epithelial-intestinal barrier (33, 34). In addition, activation of the Th17 response and development of inflammation in the colon of diabetic mice is probably related to the dysbiosis observed, since pathogenic bacteria, which we have shown to be enriched in the gut bacteriome of diabetic mice, lead to intestinal Th17 responses (35).

To confirm whether the structural and immune changes we observed in the colon of diabetic mice effectively reflected an increase in the permeability of the epithelial-intestinal barrier, we evaluated the extravasation of FITC D4000 from the intestinal lumen into the circulation as well as systemic levels of LPS. By doing so, we showed an increase in the influx of FITC D4000 from the intestinal lumen into the blood of diabetic mice compared to non-diabetic mice, indicating an increase in the epithelial-intestinal barrier permeability. Surprisingly, we noted a reduction in circulating levels of LPS in diabetic mice, even though we observed a greater diversity of Gram-negative bacteria in the gut bacteriome and a break in the epithelial-intestinal permeability. Although the exact mechanism for the reduction in the plasma LPS levels in diabetic mice remains unclear, an explanation for this phenomenon may be an increase in the LPS-binding protein (LBP) levels. In fact, LBP is increased in plasma of type 1 diabetic patients compared to non-diabetic patients (36). Another hypothesis is that LPS may be distributed to specific tissues, and, with this, its levels are reduced in diabetic mice. LPS-free is removed from the bloodstream mainly by the liver and spleen (37), therefore, we can speculate that diabetic mice may be a high tissue-specific distribution to these organs. Then, we hypothesized that LPS may be distributed to adrenals in diabetic mice. In agreement with this idea, we showed an increase in LPS levels in both the adrenal glands and colon of diabetic mice compared to non-diabetic controls. However, more experiments need to be done to understand the mechanism behind this phenomenon.

To test the hypothesis that products of the gut bacteriome of diabetic mice may be responsible for the exacerbation of glucocorticoid production by the adrenals, we treated animals with a mixture of antibiotics containing ampicillin, neomycin, and metronidazole. We chose this combination of antibiotics because it drastically reduces bacterial levels in feces and circulating levels of LPS in mice submitted to a high-fat diet (38). We showed that antibiotic therapy reduced plasma corticosterone levels and downregulated the expression of TLR4 and TRIF in the adrenal glands of diabetic mice. These data suggest that antibiotic therapy reduces LPS content in the adrenals of diabetic mice since LPS is known to induce TLR4 expression in primary hepatocytes *in vitro* (39). Our data suggest that the activation of the TLR4-TRIF pathway in the adrenals by endotoxins from the gut bacteriome is implicated in the exacerbation of corticosterone production in diabetic mice.

To understand if TLR4 activation in the adrenal glands is indeed crucial for the exacerbation of corticosterone production in diabetic mice, we used two approaches. First, we induced diabetes in C3H.HeJ mice, which are mutants of the *Lps* locus in TLR4, making them hyporesponsive to stimulation with LPS, and in C3H.He mice that is the genetic background of C3H.HeJ mice (40). Second, we treated diabetic mice with TAK-242, an antagonist of TLR4 (41). We noted that neither the treatment with TAK-242 nor the mutation of TLR4 (C3H.HeJ mice) did not modify blood glucose levels or plasma insulin levels of diabetic mice, indicating that TLR4 did not participate in alloxan-induced pancreatic β-cell injury. Nevertheless, both TLR4 mutation and pharmacological blockade decreased systemic corticosterone levels in diabetic mice, reinforcing our data indicating that TLR4 activation is crucial to the exacerbation of corticosterone production in diabetic mice. Although antibiotic therapy significantly decreased blood glucose levels in diabetic mice, this small reduction in glycemia does not seem to reduce adrenal corticosterone production in diabetic mice, since neither TLR4 mutation nor treatment with TAK-242 interfered with hyperglycemia.

To determine the mechanisms by which TLR4 activation induces the exacerbation of adrenal corticosterone production in diabetic mice, we evaluated the expression of TLR4, TRIF, and the steroidogenic machinery in the adrenals of diabetic mice after treatment with the TLR4 antagonist. We showed that treatment with TAK-242 decreased the expression of TLR4 and TRIF in the adrenal glands of diabetic mice. It is well known that LPS induces steroidogenesis in adrenocortical cells *in vitro* by a mechanism dependent on NF-κB activation (18). Although TRIF induces a MyD88-independent signaling pathway, it also activates NF-κB (42, 43), reinforcing our hypothesis that the exacerbation of adrenal corticosterone production in diabetic mice occurs via the TLR4-TRIF pathway. Nevertheless, with our data, we cannot rule out the role of other TLR4 adaptors, including MyD88, or alternative pathways such as NF-κB and IRF3 in the gut bacteria-derived LPS-TLR4 pathway-induced exacerbation of corticosterone production in diabetic animals. Finally, we also showed that TAK-242 treatment decreased expression of MC2R and 11β-HSD1 in the adrenal glands of diabetic mice. These data strongly suggest that overactivation of TLR4 in the adrenal glands of diabetic mice induces an exacerbation of corticosterone production in both a direct and an indirect ACTH-dependent way.

Our results indicate that the adrenal-gut-bacteriome axis is crucial in exacerbating adrenal steroidogenesis in diabetic mice. The mechanism involved in the increased production of corticosterone in diabetic mice depends on the up-regulation of the steroidogenic machinery in the adrenal glands induced by activation of the TLR4 pathway by endotoxins from pathogenic bacteria from the gut microbiota. With the data obtained in this study, we believe that new therapeutic strategies based on TLR4 inhibitors or prebiotics will become an essential target for treating type 1 diabetes and other diseases associated with hypercortisolism in the future.

## Data Availability

The original contributions presented in the study are included in the article/**Supplementary Material**. Further inquiries can be directed to the corresponding author. All sequences generated in this study were deposited as a Sequence Read Archive in the NCBI database with Bioproject ID PRJNA1253081 (SAMN48063965- SAMN48063969 for 16S rRNA gene of the control samples and SAMN48063970- SAMN48063974 for 16S rRNA gene for the treatment samples).
