# Supplementary material for: Gut microbiota is involved in the exacerbation of adrenal glucocorticoid steroidogenesis in diabetic animals by activation of the TLR4 pathway
Source: Front Endocrinol (Lausanne). 2025 May 27;16:1555203. doi: 10.3389/fendo.2025.1555203 (PMC12148881; doi:10.3389/fendo.2025.1555203)
Supplement: Supplementary file 1 [file DataSheet1.pdf]

Supplementary material

Results

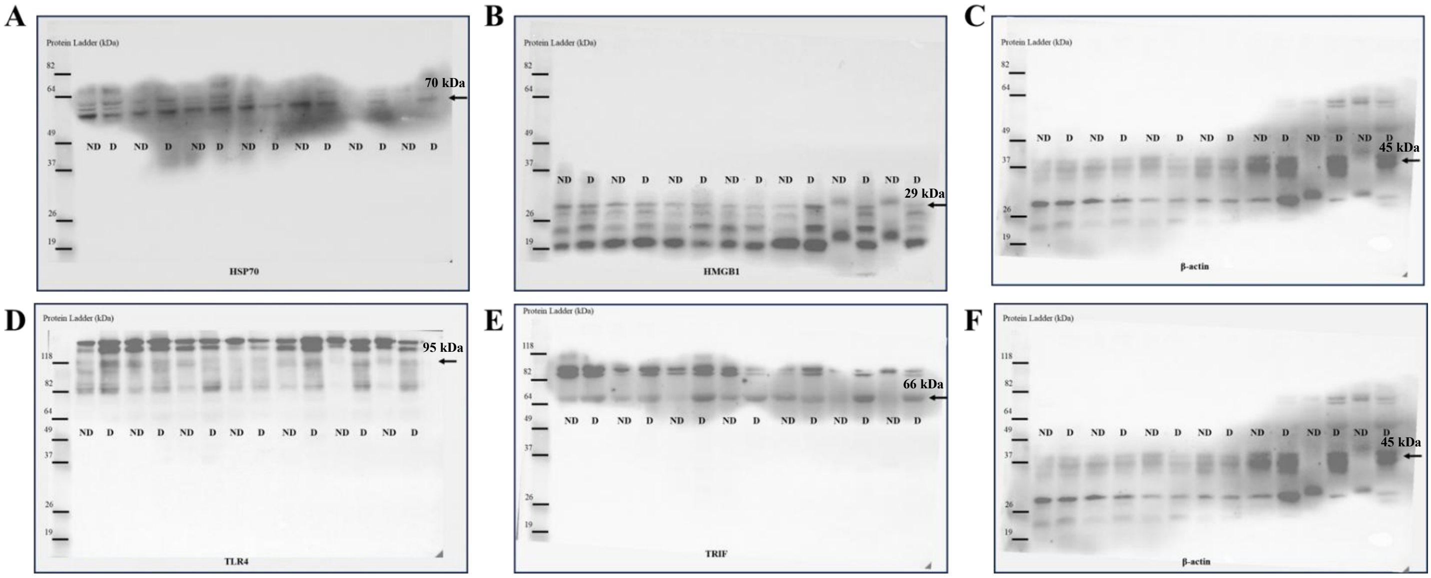

Figure S1: Western Blot evaluation of HSP70, HMGB1, TLR4, and TRIF expression in the adrenal glands of diabetic mice. Representative full-length blots of HSP70 (**A**), HMGB1 (**B**), TLR4 (**D**), TRIF (**E**), and their respective β-actin controls **(C, F**). Arrows represent the analyzed protein band. ND = Non-diabetic and D = Diabetic. HMGB1 = High mobility group box 1 protein. HSP70 = 70 kilodalton heat shock proteins. TLR4 = toll-like receptor 4. TRIF = TIR-domain-containing adapter-inducing interferon-β.

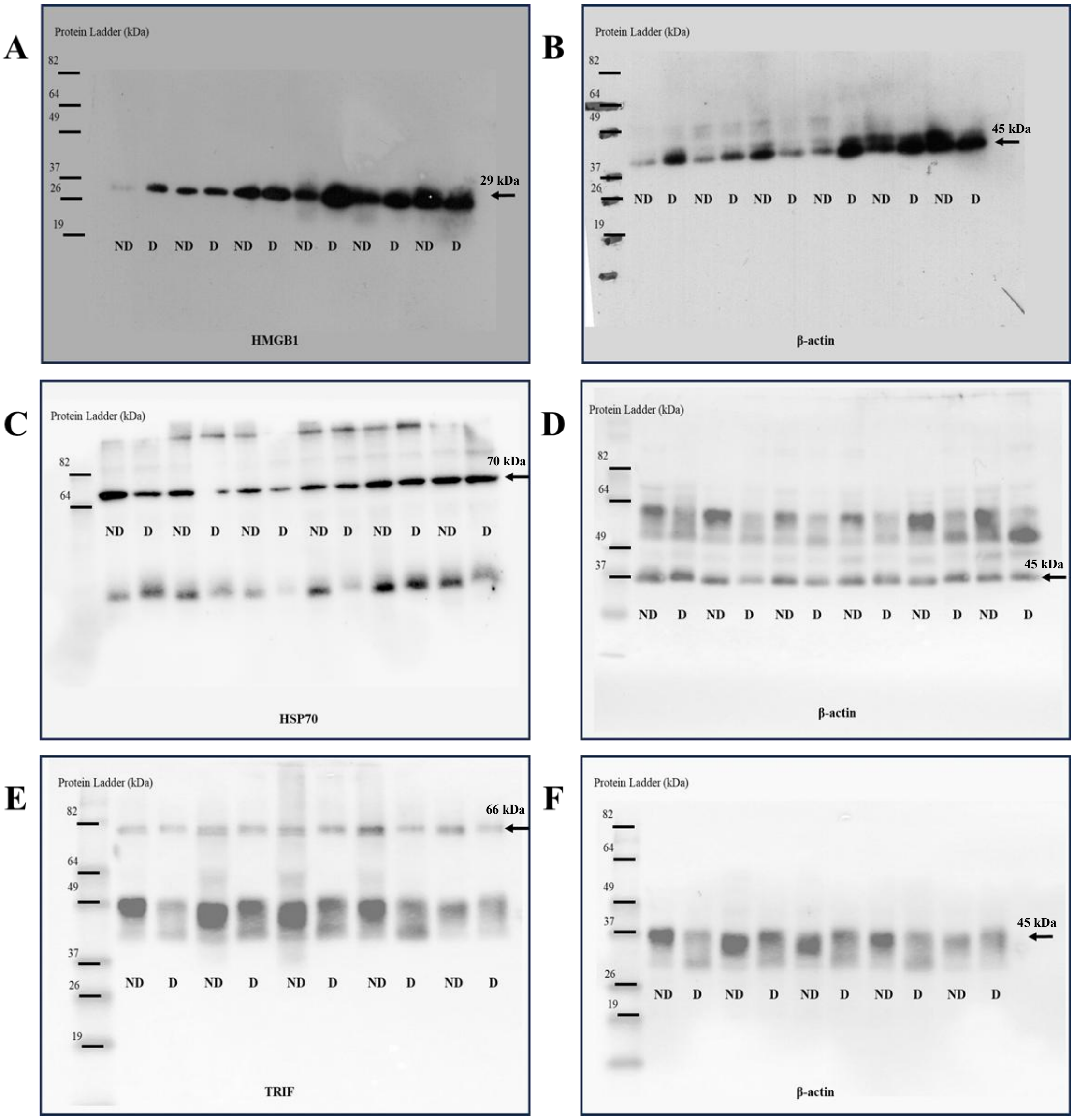

Figure S2: Western Blot evaluation of HMGB1, HSP70, and TRIF expression in the adrenal glands of diabetic rats. Representative full-length blots of HMGB1 (**A**), HSP70 (**C**), TRIF (**E**), and their respective β-actin controls (**B, D, and F**). Arrows represent the analyzed protein band. ND = Non-diabetic and D = Diabetic. HMGB1 = High mobility group box 1 protein. HSP70 = 70 kilodalton heat shock proteins. TRIF = TIR-domain-containing adapter-inducing interferon-β.

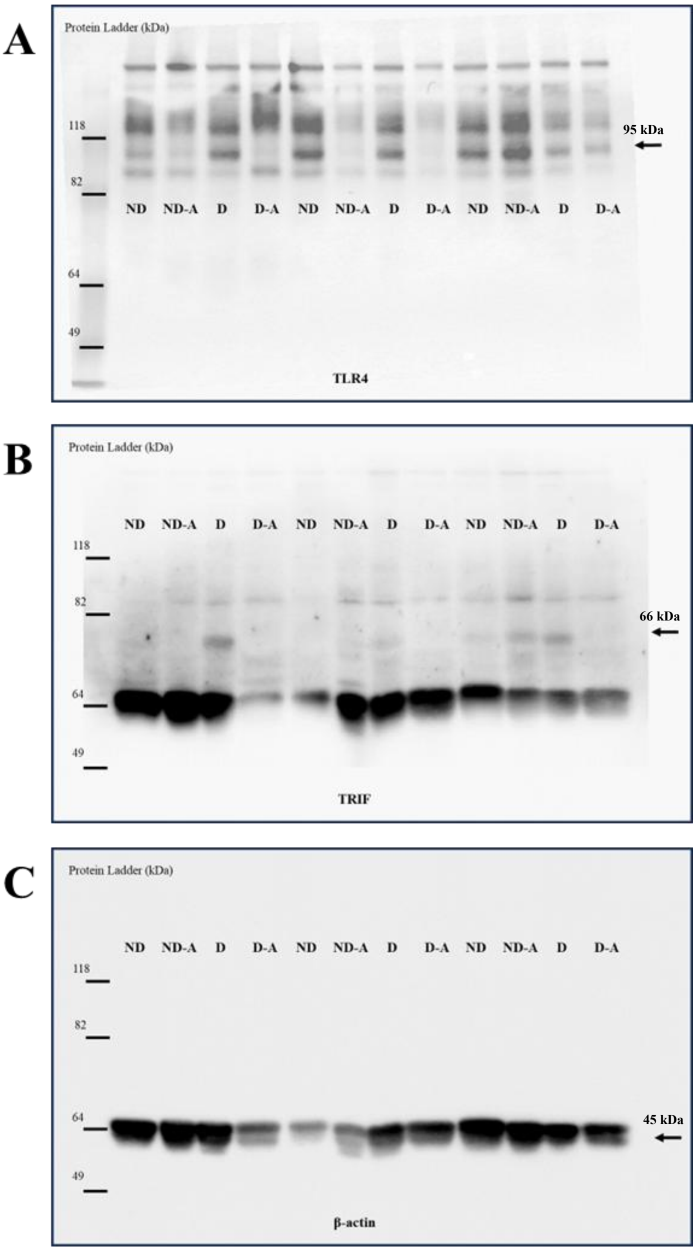

Figure S3: Western Blot evaluation of TLR4 and TRIF expression in the adrenal glands of diabetic mice treated with an antibiotic cocktail. Representative full-length blots of TLR4 (**A**), TRIF (**B**), and their respective β-actin controls **(C**). Arrows represent the analyzed protein band. ND = Non-diabetic, ND-A= non-diabetic + antibiotic, D = Diabetic, and D-A = Diabetic + antibiotic. TLR4 = toll-like receptor 4. TRIF = TIR-domain-containing adapter-inducing interferon-β.

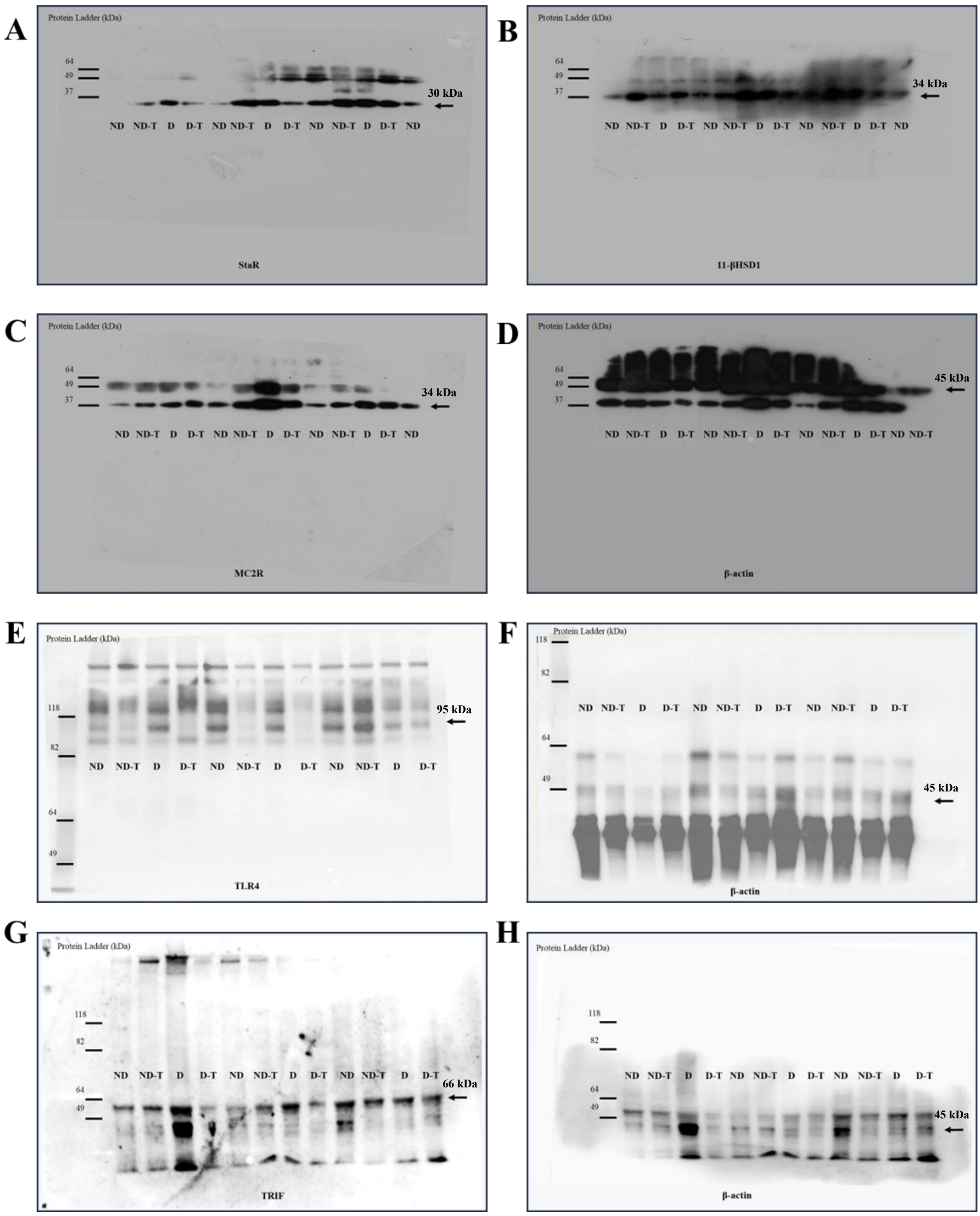

Figure S4: Western Blot evaluation of StaR, 11βHSD1, MC2R, TLR4, and TRIF expression in the adrenal glands of diabetic mice treated with TAK-242. Representative full-length blots of StaR (**A**), 11βHSD1 (**B**), MC2R (**C**), TLR4 (**E**), TRIF (**G**), and their respective β-actin controls (**D, F, H**). Arrows represent the analyzed protein band. ND = Non-diabetic, ND-T= non-diabetic + TAK-242, D = Diabetic, and D-T = Diabetic + TAK-242. 11βHSD1 = 11β-Hydroxysteroid dehydrogenase type 1. MC2R = melanocortin receptor 2. StAR = steroidogenic acute regulatory protein. TAK = TAK-242. TLR4 = toll-like receptor 4. TRIF = TIR-domain-containing adapter-inducing interferon-β.3
